# Effect of Weight Loss Surgery on Biomarkers of Angiogenesis in Obese Patients

**DOI:** 10.1007/s11695-020-04580-7

**Published:** 2020-04-19

**Authors:** Maciej Wiewiora, Anna Mertas, Marek Gluck, Alicja Nowowiejska-Wiewiora, Zenon Czuba, Jerzy Piecuch

**Affiliations:** 1 Department of General and Bariatric Surgery and Emergency Medicine, School of Medicine with the Division of Dentistry in Zabrze, Medical University of Silesia, Katowice, Poland; 2 Department of Cardiac, Vascular and Endovascular Surgery and Transplantology, Zabrze, Poland; 3 Department of Microbiology and Immunology, School of Medicine with the Division of Dentistry in Zabrze, Medical University of Silesia, Katowice, Poland; 4 Third Department of Cardiology, Silesian Centre for Heart Disease, School of Medicine with the Division of Dentistry in Zabrze, Medical University of Silesia, Katowice, Poland

**Keywords:** Angiogenesis, Obesity, Bariatric surgery

## Abstract

**Background:**

The present study aims to clarify the effects of weight loss on biomarkers associated with angiogenesis in patients who underwent laparoscopic sleeve gastrectomy (SG) or adjustable gastric banding (LAGB) in the 12-month follow-up study.

**Materials and Methods:**

We studied 24 obese patients who underwent laparoscopic weight loss surgery, 13 of whom underwent SG and 11 of whom underwent LAGB. We evaluated the circulating level of angiogenesis biomarkers preoperatively and 12 months after surgery.

**Results:**

Before surgery, the following angiogenic circulating factors were significantly higher than those of healthy subjects: angiopoietin 2 (ANG-2) (*p* < .05), granulocyte colony-stimulating factor (G-CSF) (*p* < .05), hepatocyte growth factor (HGF) (*p* < .01), platelet endothelial cell adhesion molecule (PECAM-1) (*p* < .01), and vascular endothelial growth factor (VEGF) (*p* < .05). The following angiogenesis biomarkers decreased significantly after weight loss compared with their baseline values: ANG-2 (*p* < .05), follistatin (*p* < .05), HGF (*p* < .01), PECAM-1 (*p* < .01), and VEGF (*p* < .05). There were no significant differences in the circulating levels of angiogenesis biomarkers between individuals who underwent SG and those who underwent LAGB; however, HGF, PECAM-1, and VEGF tended to be lower after SG. %BMI correlated negatively with HGF, PECAM-1, and VEGF. A similar significant negative correlation was found for %WL and %EWL. WHR correlated with PDGF-B and VEGF.

**Conclusions:**

We concluded that weight loss surgery induces the changes of circulating levels of angiogenesis biomarkers in obese patients. The changes in angiogenesis status in obese patients who lost weight after bariatric surgery depended on the amount of weight loss.

## Introduction

Obesity induces adipose tissue dysfunction that typically leads to the overexpression of proinflammatory adipokines, decreased expression of anti-inflammatory adipokines, and changes in adipose tissue vasculature, depending on factors that affect angiogenesis. There are a large number of factors involved in regulating angiogenesis in adipose tissue that depend on the complicated relationships between angiogenic and angiostatic factors [1–3]. Adipose tissue is actively involved in angiogenesis through the secretion of biologically active proangiogenic factors such as adiponectin, leptin, vascular endothelial growth factor (VEGF) [4], platelet-derived growth factor receptor (PDGFR) [5], and hepatocyte growth factor (HGF) [6], although there are differences in the impact of angiogenesis on the directions of physiological changes between white and gray adipose tissue [7]. Adipose tissue expansion is critically dependent on adipose vasculature, which is modified by several mechanisms, but the balance between angiogenic factors and their inhibitors plays a principal role in the plasticity of the adipose vascular network [8]. Another study highlighted the role of shear stress in controlling angiogenic origination and offered a potential homeostatic mechanism for regulating vascular density [9, 10]. There is evidence that adipose tissue is actively involved in angiogenesis through the secretion of biologically active substances, including angiogenic and angiostatic factors [11, 12]. Physiological angiogenes are necessary to correct the functions of some organs, but an altered mechanism involving angiogenesis leads to the formation of new blood vessels and contributes to various diseases, including malignant, ischemic, and inflammatory disorders, which are associated with obesity [13]. The relationship of adipogenesis with angiogenesis in a dietary-induced obesity animal model is associated with extensive neovascularization, not only locally in fat but also in other organ systems, which can be the base of the pathophysiological mechanism of the most frequent obesity-related comorbidities. Previously, we confirmed that obese subjects are associated with high levels of proangiogenic circulating biomarkers in the blood [14]. In this study, we evaluated the impact on the venous hemodynamics of the femoral vein on angiogenesis status in morbidly obese patients and confirmed that circulating angiogenesis biomarkers were significantly higher in the obese patients compared with the control group. The present study aims to clarify the effects of weight loss on biomarkers associated with angiogenesis in patients who underwent laparoscopic sleeve gastrectomy (SG) or adjustable gastric banding (LAGB) in the 12-month follow-up study.

## Materials and Methods

We studied 24 morbidly obese patients who underwent SG or LAGB. There were 6 males and 18 females in the study, with a mean age of 38.4 ± 8.8 years, a mean weight of 132.2 ± 21.4 kg, and a mean body mass index (BMI) of 45.6 ± 5.9 kg/m^2^. Exclusion criteria included smoking; diabetes mellitus; thyroid disease; chronic kidney disease; anemia; abnormal coagulation parameters; uncontrolled hypertension; history of leg vein thrombosis; history of miscarriage; antithrombotic, estrogen, or/and contraceptive therapy; clinical signs of arterial disease; and chronic venous signs. The control group consisted of 15 nonobese people without arterial hypertension, diabetes mellitus, or any of the other abovementioned features. The study protocol was accepted by the ethical committee of the Medical University of Silesia and all participants provided written consent.

### Study Design

The main purpose of the study is the assessment of the impact of weight loss after surgery on directional changes in circulating biomarkers of angiogenesis. Patients selected either LAGB or SG based on their personal preferences after a detailed and objective consultation with the surgeon. Patients were followed for 1 year following the procedure. The patients were weighed, examined, and interviewed by the surgeon preoperatively and at 1, 6, and 12 months after the operation. Blood was drawn for angiogenesis measurements preoperatively and at 12 months after the operation. Data from morbidly obese subjects were compared with those from the control group before and after surgery. We assume that the angiogenesis status before surgery in the obese population differed from that in the healthy control group. Given that the normalization of angiogenesis is based on evaluated angiogenesis biomarkers in the obese group, we assumed a lack of differences at 12 months after the operation compared with the control group. The effects of the two surgeries on angiogenesis were also assessed.

### Angiogenesis Measurements

Blood samples were collected from the cubital vein with a syringe for an angiogenesis examination preoperatively and 12 months after the operation. The Bio-Plex Pro™ Human Angiogenesis 9-plex kit (Bio-Rad Laboratories Inc., Hercules, CA, USA) was used to measure the following angiogenesis parameters: angiopoietin-2 (ANG-2), follistatin, granulocyte colony-stimulating factor (G-CSF), HGF (hepatocyte growth factor), platelet-derived growth factor-B (PDGF-BB), platelet endothelial cell adhesion molecule (PECAM-1), and VEGF in serum samples of patients from the study and control groups.

The angiogenesis parameters were quantitated using the Bio-Plex 200 System based on xMAP suspension array technology (Bio-Rad Laboratories Inc., Hercules, CA, USA). The immunoassay was designed for the multiplexed quantitative measurement of multiple parameters in a single well using 50-μl samples. All procedures were followed according to the manufacturer’s manual. Standard curves for each studied parameter were performed using standard solutions with known concentrations of measured angiogenesis parameters or cytokines. The serum samples were diluted fourfold in sample diluent. The standards, controls, and serum samples were incubated with antibody-conjugated beads for 30 min. Next, after washing with buffer, detection antibodies were added to each well for 30 min. After washing with buffer to remove the unbound detection antibodies, streptavidin-PE was added to each well for 30 min. Then, after washing to remove the unbound streptavidin-PE, the beads bound to each angiogenesis parameter or studied cytokine were analyzed in the Bio-Plex Array Reader in Bio-Plex 200 System. The intensity of fluorescence was evaluated using Bio-Plex Manager software from the Bio-Plex 200 System.

### Surgery Procedure

Patients enrolled in the study underwent SG or LAGB. SG was performed according to the commonly used technique [15]. The LAGB procedure was employed according to the pars flaccida technique, which is known to be associated with better outcomes than the perigastric technique [16].

### Statistical Analysis

Continuous variables are presented as the means ± SDs or as medians with interquartile ranges if they were not normally distributed. Categorical variables are presented as absolute numbers and percentages. The Shapiro-Wilk test was used for all continuous variables to test for a normal distribution. Statistical comparisons were performed using the unpaired Student’s *t* test and the *U* Mann-Whitney test for nonnormally distributed data. Fisher’s exact test was performed to compare differences in categorical data. Comparisons of each dependent variable before surgery and 12 months after surgery were assessed using the paired Student’s *t* test and the paired Wilcoxon nonparametric test for nonnormally distributed data. The associations between continuous variables were tested using Spearman’s rank correlation. The statistical analysis was performed using Statistica 12 (StatSoft, Inc. Tulsa, OK, USA).

## Results

**Fig. 1:**
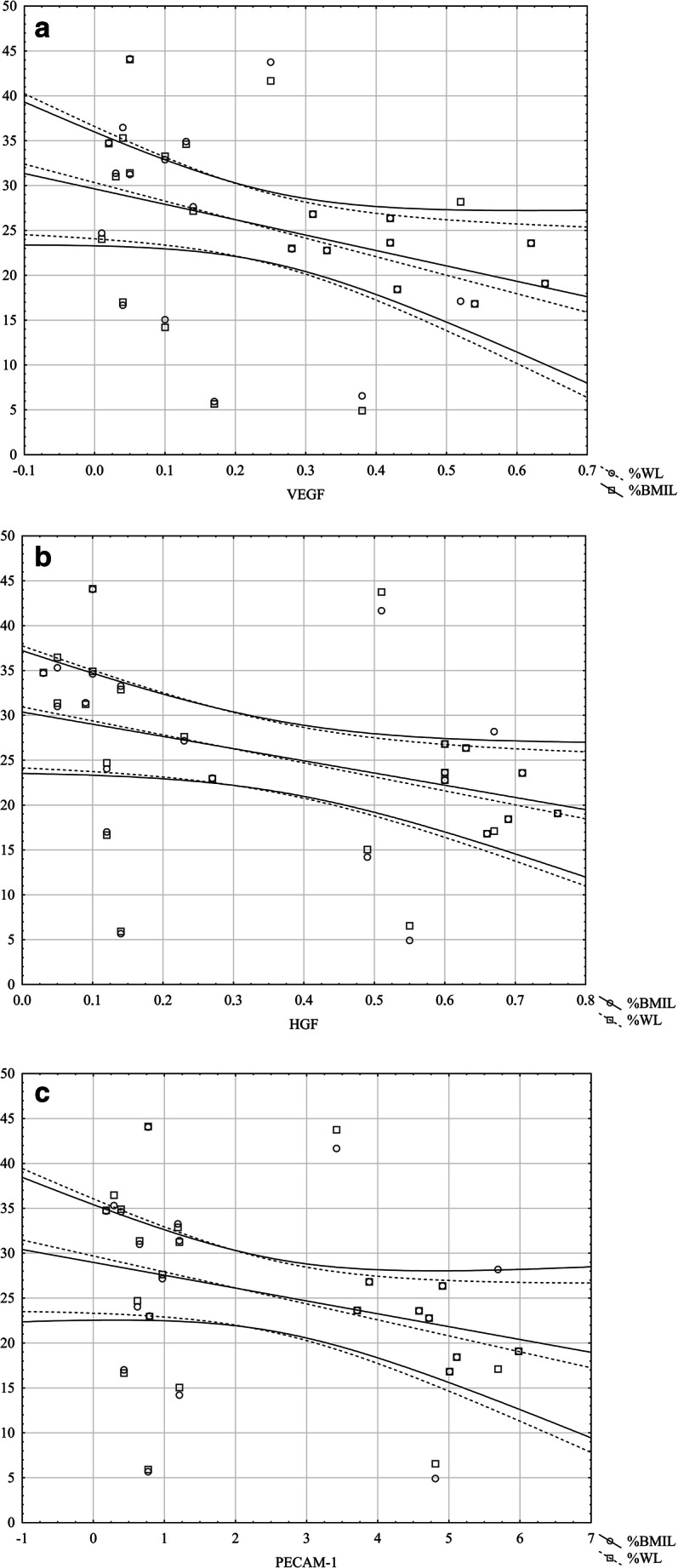
Correlation between angiogenesis vs %WL and %BMI 12 months after surgery. **a** With VEGF. **b** With HGF. **c** With PECAM-1

**Fig. 2:**
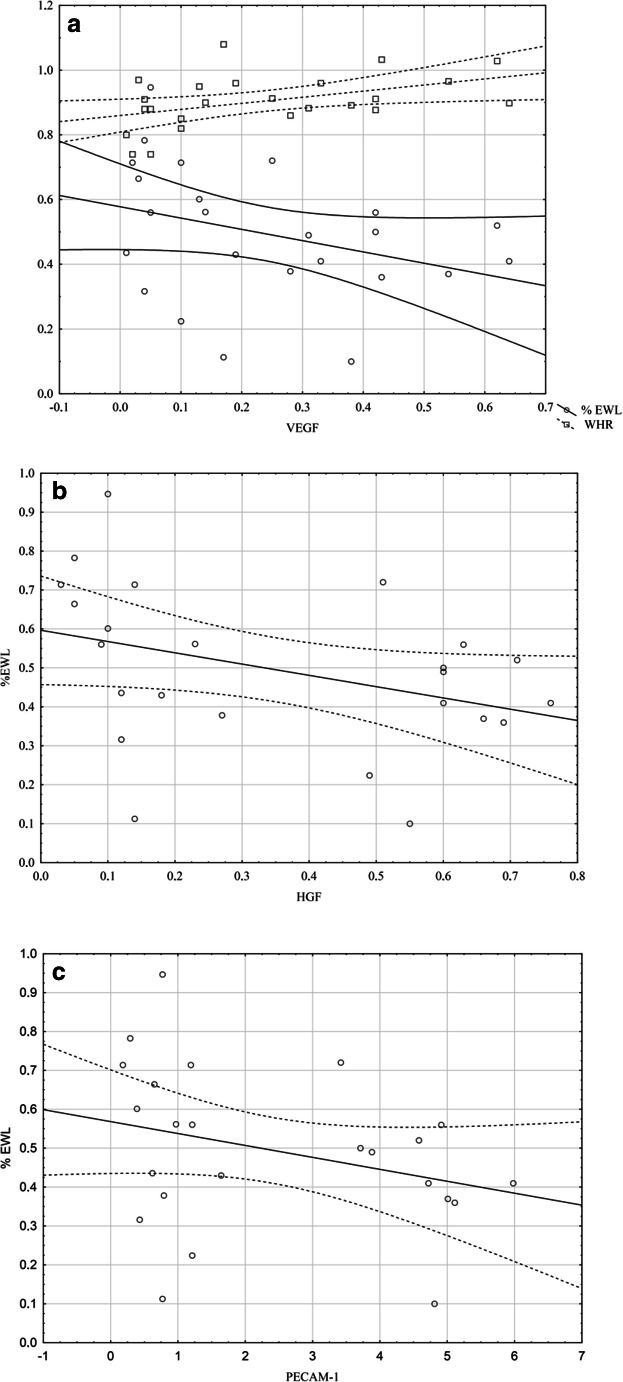
Correlation between angiogenesis vs %EWL and WHR 12 months after surgery. **a** With VEGF. **b** With HGF. **c** With PECAM-1

The baseline characteristics of the study population are presented in Table 1. The sex distribution and ages were similar in the obese and control groups. As expected, the anthropometric parameters were higher in the obese subjects. There were significant differences in circulating angiogenesis parameters in the obese group compared with those in the control group (Table 1). All of the angiogenesis parameters were higher in obese subjects, but significant differences were observed for ANG-2 (*p* < .05), G-CSF (*p* < .05), HGF (*p* < .01), PECAM-1 (*p* < .01), and VEGF (*p* < .01). The anthropometric and angiogenesis parameters were different at 12 months after surgery, and the findings are presented in Table 2. All angiogenesis biomarkers decreased 12 months postoperatively. Significant differences were observed for ANG-2, in which the median declined from 1.35 pg/ml at baseline to 0.53 pg/ml 12 months after surgery (*p* < .05), follistatin from 0.24 to 0.15 pg/ml (*p* < .05), HGF from 0.7 to 0.27 pg/ml (*p* < .01), PECAM-1 from 5.5 to 1.21 pg/ml (*p* < .01), and VEGF from 0.35 to 0.14 pg/ml (*p* < .05). The G-CSF and PDGF-B changes were not significant. No differences were found in any angiogenesis parameters 12 months postoperatively in the obese group compared with those in the control group, except PECAM-1 and VEGF. Twelve months after surgery, the percentage of weight loss (%WL) was 25 ± 8, the percentage of BMI loss (%BMIL) was 25.6 ± 9, and the percentage of excess weight loss (%EWL) was 48.9 ± 2. The comparison of the SG and LAGB patients revealed no significant differences between circulating angiogenic biomarkers (Table 3). The correlations between angiogenesis parameters and anthropometric changes at 12 months after surgery are presented in Table 4. %BMIL correlated negatively with HGF, PECAM-1, and VEGF (Fig. 1). A similar significant negative correlation was found for %WL (Fig. 1) and %EWL (Fig. 2). Waist-hip ratio (WHR) correlated with PDGF-B and VEGF (Fig. 2). No correlations were found between the postoperative anthropometric indices and ANG-2, follistatin, or G-CSF.

**Table 1:** Baseline characteristics of obese patients and control participants

		Morbid obesity *N* = 24	Control *N* = 15	*p* value
Ages (years)		38.4 ± 8.8	33.6 ± 9.3	0.1
Gender	Male	6 (25%)	5 (33.3%)	0.7
	Female	18 (75%)	10 (66.6%)	0.5
Weight (kg)		132.2 ± 21.4	70.2 ± 10.2	< .01
BMI (kg/m^2^)		45.6 ± 5.9	23.4 ± 1.4	< .01
WHR		0.9 (0.8–1)	0.8 (0.8–0.9)	< .01
ANG-2 (pg/ml)		1.3 (0.5–1.5)	0.3 (0.1–0.6)	< .05
Follistatin (pg/ml)		0.2 (0.1–0.4)	0.1 (0.1–0.2)	0.05
G-CSF (pg/ml)		0.07 (0.05–0.08)	0.05 (0.04–0.06)	< .05
HGF (pg/ml)		0.7 (0.3–0.8)	0.1 (0.07–0.3)	< .01
PDGF-BB (pg/ml)		3.5 (2–3.9)	2.1 (1.4–3.3)	0.06
PECAM-1 (pg/ml)		5.5 (0.8–6.6)	0.4 (0.3–0.9)	< .01
VEGF (pg/ml)		0.3 (0.1–0.5)	0.07 (0.06–0.1)	< .01

Data are presented as the means ± SDs or medians (interquartile ranges) for continuous variables. Group differences were calculated using the unpaired Student’s *t* test or the Mann-Whitney *U* test for nonnormally distributed data. *BMI*, body mass index; *WHR*, waist-to-hip circumference ratio; *ANG-2*, angiopoietin 2; *G-CSF*, granulocyte colony-stimulating factor; *HGF*, hepatocyte growth factor; *PDGF-BB*, platelet-derived growth factor-BB; *PECAM-1*, platelet endothelial cell adhesion molecule; *VEGF*, vascular endothelial growth factor

**Table 2:** Changes in each variable at 12 months after surgery

	Baseline *N* = 24	12 months *N* = 24	*p* value
Weight (kg)	132.2 ± 21.4	96.7 ± 12.4	< .01
BMI (kg/m^2^)	45.6 ± 5.9	33.7 ± 3.9	< .01
WHR	0.92 (0.8–1)	0.89 (0.8–0.9)	< .01
ANG-2 (pg/ml)	1.3 (0.5–1.5)	0.5 (0.2–1.3)	< .05
Follistatin (pg/ml)	0.2 (0.1–0.4)	0.1 (0.1–0.2)	< .05
G-CSF (pg/ml)	0.07 (0.05–0.08)	0.06 (0.05–0.07)	0.2
HGF (pg/ml)	0.7 (0.3–0.8)	0.2 (0.1–0.6)	< .01
PDGF-BB (pg/ml)	3.5 (2–3.9)	3.1 (1.5–3.4)	0.1
PECAM-1 (pg/ml)	5.5 (0.8–6.6)	1.21 (0.7–4.7)	< .01
VEGF (pg/ml)	0.3 (0.1–0.5)	0.14 (0.1–0.38)	< .05

Data are presented as the means ± SDs or medians (interquartile ranges) for continuous variables. Group differences were calculated using the paired Student’s *t* test or Wilcoxon’s test for nonnormally distributed data. For abbreviations, see Table 1

**Table 3:** Differences in angiogenesis variable 12 months after surgery depend on the operation type

	ANG-2	Follistatin	G-CSF	HGF	PDGF-BB	PECAM-1	VEGF
BMI	− 0.1	− 0.3	0.07	− 0.2	− 0.3	− 0.1	− 0.2
WHR	0.1	− 0.1	0.3	0.3	0.4*	0.3	0.5*
%WL	− 0.1	− 0.03	− 0.2	− 0.5*	− 0.2	− 0.5*	− 0.5*
%BMI	− 0.2	− 0.04	− 0.2	− 0.5*	− 0.2	− 0.4*	− 0.4*
%EWL	− 0.1	− 0.2	− 0.2	− 0.5*	− 0.2	− 0.4*	− 0.4*

Data are presented as the median (interquartile range). Group differences were calculated using the Mann-Whitney *U* test. *SG*, laparoscopic sleeve gastrectomy; *LAGB*, laparoscopic adjustable gastric banding; for other abbreviations, see Table 1

**Table 4:** Correlation between angiogenesis parameters and anthropometric changes at 12 months after surgery

	ANG-2	Follistatin	G-CSF	HGF	PDGF-BB	PECAM-1	VEGF
BMI	− 0.1	− 0.3	0.07	− 0.2	− 0.3	− 0.1	− 0.2
WHR	0.1	− 0.1	0.3	0.3	0.4*	0.3	0.5*
%WL	− 0.1	− 0.03	− 0.2	− 0.5*	− 0.2	− 0.5*	− 0.5*
%BMI	− 0.2	− 0.04	− 0.2	− 0.5*	− 0.2	− 0.4*	− 0.4*
%EWL	− 0.1	− 0.2	− 0.2	− 0.5*	− 0.2	− 0.4*	− 0.4*

## Discussion

The prevalence of obesity worldwide has increased rapidly over the last three decades. While there are differences according to regions and countries, globally, an increased prevalence of obesity has been observed across obesity classes and young populations [17]. Weight loss surgery has become the most popular method to treat morbidly obese patients because it has substantial beneficial associations with weight loss and several clinical outcomes [18].

The recent studies showed that obesity-associated weight loss via diet contributed to the reduction in some circulating levels of positive regulators of angiogenesis [19, 20], depending on the extent of weight loss [19]. Other authors demonstrated that PDGF-B-PDGFRβ signaling regulates adipose tissue expansion and glucose metabolism via vascular remodeling in an animal model of diet-induced obesity [5]. Deletion of the PDGF or PDGFRβ genes inhibited neovascularization in adipose tissue and improved inflammation in adipose tissue and glucose metabolism.

However, the impacts of weight loss surgery on the biomarkers associated with angiogenesis in obese patients have been poorly defined. Recent studies have established the effect of SG on angiogenesis but only in obese animal models of T2D (type 2 diabetes) [21]. This study showed that weight loss related to SG improved angiogenesis and adipose tissue vascular function. Serum adiponectin and adipose tissue levels of perilipin and PPAR-γ, which are regulators of angiogenesis, were also improved after surgery. In humans, it was shown that SG influences the expression of ANG and its receptor Tie-2 in adipose tissue, and this modification causes an anti-inflammatory effect [22]. The authors concluded that bariatric surgery modifies the proangiogenic profile and reduces angiogenic expression in the circulation and adipose tissue. ANG-2 is considered a proangiogenic factor; however, its role in the angiogenic process has not yet been elucidated. A recent study demonstrated that ANG-2 overexpression in white adipose tissue in an animal model increased adipose tissue vascularization and improved metabolic status [23]. The interaction between VEGF and ANG-2 plays an important functional role in the regulation of physiological and pathological angiogenesis, but the specific participation of ANG in adipose tissue physiology is poorly defined [24].

The study examines the effects of weight loss due to bariatric surgery on comprehensive evaluations of circulating levels of angiogenesis biomarkers. We assess the impact of significant weight loss after surgery, not the trends of changes during the postoperative period. To show the direction of change, measurements of angiogenesis parameters and clinical and anthropometric tests were performed before surgery and 12 months after surgery and were compared with those of the control group. The study was conducted in a group of 24 obese patients who underwent SG or LAGB. The publication of research results based on small groups of subjects is permissible and has been practiced, provided, of course, that several conditions are met. The methodology of such a study should be reproducible and carried out according to a standardized research model. Statistical methods must be chosen appropriately, and the statistical analysis must be carried out using a credible instrument. A very important element, especially in small study groups, is the appropriate selection of the study group, the degree of statistical significance of individual results, and the reproducibility of the results. A small group of subjects may be insufficient to assess the interrelations between the studied parameters, such as in uni- or multivariate regression; therefore, these analyses have not been performed. All patients were subjected to a prospective postoperative control according to a homogeneous, standardized research model. The factors used as exclusion criteria included those that can potentially influence the observed angiogenesis parameters. We tried to create a select group of obese patients to potentially ignore the influence of factors other than weight loss. Considering the difficulties of the selection of obese patients before bariatric surgery, with numerous exclusion criteria and the precision of statistical methods, a study group consisting of 24 subjects seems to be sufficient.

The results confirmed that obesity is associated with the overexpression of angiogenic factors, and all proangiogenic circulating biomarkers were higher in obese individuals compared with the levels in healthy subjects. Previously, we performed two studies concerning angiogenesis in obese subjects in a large group of participants. Despite the difference in the sizes of the study groups, the results concerning angiogenesis status were very similar, and obese patients had elevated levels of angiogenesis markers compared with controls [14, 25].

We also found that weight loss surgery changed the proangiogenic profile, leading to a reduction in all angiogenesis biomarkers in obese patients 12 months after the operation. We observed a significant decrease in all angiogenesis biomarkers, except G-CSF and PDGF-BB, compared with the baseline values. There were no differences in angiogenesis status between the SG and LAGB procedures; however, HGF, PECAM-1, and VEGF tended to be lower after SG. Our findings also suggest that the beneficial effects of bariatric surgery on angiogenesis are associated with obesity depending on the amount of weight loss. This relationship confirmed a correlation between angiogenesis parameters and anthropometric changes 12 months after surgery. We presented the correlation between angiogenesis parameters and anthropometric changes at 12 months after surgery in all groups. We did not perform correlation analysis in either group (SG and LAGB) because the assumption of the study was to assess the effect of weight reduction, not the type of surgery; such analysis requires a separate study. Indeed, subanalysis in the subgroups (SG and LAGB) could be unreliable due to the small number of participants in each subgroup.

Adipose tissue, especially visceral adipose tissue, is responsible for the production of many paracrine and endocrine substances, including those that are actively involved in angiogenesis. These substances may affect the function of other organs and tissues in the human body through various complex mechanisms. Expansion of adipose tissue in the context of diet-induced obesity leads to hyperplasia and hypertrophy of adipocytes and is associated with extensive neovascularization, including microvessel growth and vascular network remodeling. This process is critically dependent on adipose vasculature, which is modified by the balance between angiogenic factors and angiogenic inhibitors. Adequate angiogenesis is necessary for adipose tissue enlargement because it requires efficient blood perfusion for supplementation of nutrition and oxygen in newly enlarged areas. A recent study has shown that adequate angiogenesis improved inflammatory and metabolic status and modulated insulin sensitivity [26]. On the other hand, enlarged adipocytes develop relative tissue hypoxia that leads to inflammation and angiogenesis and the further enlargement of adipocytes. Adipose tissue expansion is critically dependent on adipose vasculature, which is modified by several mechanisms, but the balance between angiogenic factors and their inhibitors plays a principal role in the plasticity of the adipose vascular network [3, 27, 28].

There are also studies that have shown that in animal models, the inhibition of angiogenesis, such as endostatin, effectively protects diet-induced obesity and related metabolic disorders [29]. Ya, A. et al. demonstrated that ANG-2 overexpression in white adipose tissue in an animal model increased adipose tissue vascularization, improved metabolic status, and exerted an anti-inflammatory effect, but the specific participation of ANG in adipose tissue physiology is poorly defined [23]. On the other hand, data show that adipose tissue–associated angiogenesis is caused by hypoxia associated with decreased capillary density and, consequently, decreased blood flow. Therefore, the effect of angiogenesis on tissue blood flow is not one-sided, as the hemodynamic consequences of angiogenesis can affect the amount of tissue perfusion resulting from several interrelated mechanisms.

In our study, we detected circulating angiogenesis biomarkers and their changes 12 months after surgery. A high level of angiogenesis biomarkers may suggest the activation of angiogenesis, but we do not speculate about the genesis of this process and the induction of inflammatory mediator release. Angiogenic activity in obese individuals may be one of the physiological consequences of progressive obesity as an adaptive mechanism to changes in blood flow in the microcirculation network. The reason for this angiogenesis status in obese individuals may be different. We only presented the effects of surgery on angiogenesis and did not speculate about the pathophysiological mechanisms associated with high levels of circulating angiogenesis biomarkers.

## Conclusions

We concluded that weight loss surgery induces the changes of circulating levels of angiogenesis biomarkers in obese patients. The changes in angiogenesis status in obese patients who lost weight after bariatric surgery depended on the amount of weight loss. No differences were found in any angiogenesis parameters 12 months postoperatively in the obese group compared with those in the control group, which may indicate a beneficial direction of changes, but further study is required to demonstrate whether these changes in circulating angiogenesis biomarkers improve obesity-associated disturbances and adipose tissue health. Knowledge of the mechanism of angiogenesis and the disturbance of the mechanism involved in physiological angiogenesis is still rudimentary. Enhanced angiogenesis does not always indicate pathological processes, and changes in circulating angiogenesis related to some degree of increased fat mass are physiological consequences of sedentary behavior and may be an adaptive mechanism.
